# Potential Application of Digitally Linked Tuberculosis Diagnostics for Real-Time Surveillance of Drug-Resistant Tuberculosis Transmission: Validation and Analysis of Test Results

**DOI:** 10.2196/medinform.9309

**Published:** 2018-02-27

**Authors:** Kamela Charmaine Ng, Conor Joseph Meehan, Gabriela Torrea, Léonie Goeminne, Maren Diels, Leen Rigouts, Bouke Catherine de Jong, Emmanuel André

**Affiliations:** ^1^ Mycobacteriology Unit Department of Biomedical Sciences Institute of Tropical Medicine Antwerp Belgium; ^2^ Pôle de Microbiologie Médicale Institut de Recherche Expérimentale et Clinique Université Catholique de Louvain Brussels Belgium; ^3^ Department of Biomedical Sciences University of Antwerp Antwerp Belgium; ^4^ Laboratory of Clinical Bacteriology and Mycology Katholieke Universiteit Leuven Leuven Belgium

**Keywords:** tuberculosis, drug resistance, rifampicin-resistant tuberculosis, rapid diagnostic tests, Xpert MTB/RIF, Genotype MTBDRplus v2.0, Genoscholar NTM + MDRTB II, RDT probe reactions, rpoB mutations, validation and analysis, real-time detection

## Abstract

**Background:**

Tuberculosis (TB) is the highest-mortality infectious disease in the world and the main cause of death related to antimicrobial resistance, yet its surveillance is still paper-based. Rifampicin-resistant TB (RR-TB) is an urgent public health crisis. The World Health Organization has, since 2010, endorsed a series of rapid diagnostic tests (RDTs) that enable rapid detection of drug-resistant strains and produce large volumes of data. In parallel, most high-burden countries have adopted connectivity solutions that allow linking of diagnostics, real-time capture, and shared repository of these test results. However, these connected diagnostics and readily available test results are not used to their full capacity, as we have yet to capitalize on fully understanding the relationship between test results and specific *rpoB* mutations to elucidate its potential application to real-time surveillance.

**Objective:**

We aimed to validate and analyze RDT data in detail, and propose the potential use of connected diagnostics and associated test results for real-time evaluation of RR-TB transmission.

**Methods:**

We selected 107 RR-TB strains harboring 34 unique *rpoB* mutations, including 30 within the rifampicin resistance–determining region (RRDR), from the Belgian Coordinated Collections of Microorganisms, Antwerp, Belgium. We subjected these strains to Xpert MTB/RIF, GenoType MTBDR*plus* v2.0, and Genoscholar NTM + MDRTB II, the results of which were validated against the strains’ available *rpoB* gene sequences. We determined the reproducibility of the results, analyzed and visualized the probe reactions, and proposed these for potential use in evaluating transmission.

**Results:**

The RDT probe reactions detected most RRDR mutations tested, although we found a few critical discrepancies between observed results and manufacturers’ claims. Based on published frequencies of probe reactions and RRDR mutations, we found specific probe reactions with high potential use in transmission studies: Xpert MTB/RIF probes A, Bdelayed, C, and Edelayed; Genotype MTBDR*plus* v2.0 WT2, WT5, and WT6; and Genoscholar NTM + MDRTB II S1 and S3. Inspection of probe reactions of disputed mutations may potentially resolve discordance between genotypic and phenotypic test results.

**Conclusions:**

We propose a novel approach for potential real-time detection of RR-TB transmission through fully using digitally linked TB diagnostics and shared repository of test results. To our knowledge, this is the first pragmatic and scalable work in response to the consensus of world-renowned TB experts in 2016 on the potential of diagnostic connectivity to accelerate efforts to eliminate TB. This is evidenced by the ability of our proposed approach to facilitate comparison of probe reactions between different RDTs used in the same setting. Integrating this proposed approach as a plug-in module to a connectivity platform will increase usefulness of connected TB diagnostics for RR-TB outbreak detection through real-time investigation of suspected RR-TB transmission cases based on epidemiologic linking.

## Introduction

Tuberculosis (TB) has the highest mortality of any infectious disease and is the principal cause of death related to antimicrobial resistance [1]. Efforts to control TB are continuously hampered by complex regimens and low treatment success of drug-resistant cases [1,2]. Multidrug-resistant TB (MDR-TB), defined as resistance to the first-line drugs rifampicin and isoniazid, remains an urgent public health crisis, as only about 39% of notified, confirmed, and previously treated people with TB were tested for rifampicin resistance in 2016, and only 1 in 5 received treatment, of whom only half were cured [1].

Rifampicin resistance is an epidemiologically and clinically important surrogate marker for MDR-TB, as most rifampicin-resistant strains are also resistant to isoniazid [2-4]. Rifampicin resistance-conferring mutations are primarily situated at codon positions 426 to 452 [2] within the 81-bp rifampicin resistance–determining region (RRDR) of the *Mycobacterium tuberculosis* RNA polymerase β subunit (*rpoB*) gene [4-6]. Consequently, commercially available molecular tests developed for rapid detection of MDR-TB only capture mutations in the RRDR, with or without mutations associated with isoniazid resistance [2,3,6].

Recognizing the immediate need to rapidly detect rifampicin-resistant TB (RR-TB), the World Health Organization has recommended implementation of the following rapid diagnostic tests (RDTs) as primary tools for detection: Xpert MTB/RIF (Cepheid, Sunnyvale, CA, USA), GenoType MTBDR*plus* v2.0 (Hain Lifescience GmbH, Nehren, Germany), and Genoscholar NTM + MDRTB II (NIPRO Corporation, Osaka, Japan). Xpert MTB/RIF is the most widely deployed RDT globally, implemented as the initial diagnostic tool by 28 out of 48 high-burden countries for patients with pulmonary TB symptoms by the end of 2016 [1]. It uses real-time polymerase chain reaction and molecular beacon technology, involving probes specifically binding to wild-type sequences, whereas the other RDTs are line probe assays, which rely on hybridization and comprise both wild-type and mutant probes. The specific features and limitations of the RDTs, along with key recommendations from the World Health Organization, have been previously described [6]. The global integration and scale-up of these RDTs in TB diagnostics has dramatically improved detection of MDR-TB [1,4,6-8].

Large gaps in detection and treatment of RR-TB can result in resistant strains circulating within populations [7,9]. Particularly in high-burden settings, transmission was found to be the predominant cause of the globally rising rates of RR-TB and MDR-TB [8,10,11], with an estimated 600,000 new cases of RR-TB arising in 2016, of which 490,000 were MDR-TB [1,8]. To achieve the global targets of ending TB by 2030 [1], there is an urgent need to shift from the current inefficient paper-based investigation of drug-resistant cases to fully digitized surveillance allowing for real-time detection of transmission hotspots.

When Xpert MTB/RIF was rolled out in 2010, there was no system in place to systematically extract, interpret, and employ test results for surveillance. Accumulating data stored in local machines were clearly underused; portions of data even get corrupted and are not used at all. For a few years after, the gap in TB diagnostics implementation was addressed by the emergence of diagnostic eHealth solutions, particularly the development of connectivity solutions. Xpert MTB/RIF machines are now being linked, along with generated test results, to connectivity platforms such as DataToCare (Savics, Brussels, Belgium) and GxAlert (SystemOne, Springfield, MA, USA). These connectivity platforms automate the collection of diagnostic test results from each health facility in a particular setting. They consolidate all data into a built-in analytics system that automates extraction of useful information from raw test data. Generated information is then securely shared with different stakeholders—clinicians, national TB control programs, and patients—through text messaging, email, or a national database [12].

Despite this technological advancement, readily available and accumulating TB diagnostic test results have not been used to date for disease surveillance, due to the limited understanding of the correlation between RDT probe reactions and specific *rpoB* mutations. In accordance with the consensus of global TB experts on the potential of diagnostic connectivity for TB elimination published in 2016 [12], we believe that RDT results coupled with appropriate analysis may present crucial clinical and public health information that could be employed as a molecular epidemiologic tool in field conditions to trace RR-TB transmission hotspots in high-burden TB settings. They could also aid in resolving the discordance between phenotypic drug susceptibility testing and RDT results, primarily for disputed mutations, which are often missed in *Mycobacterium* growth indicator tube phenotypic drug susceptibility testing [13]. In this work, we aimed to analyze RDT data in detail, visualize test results representing specific RRDR mutations, and propose a novel approach of fully using digitally linked TB diagnostics and readily available test results for real-time monitoring of RR-TB transmission.

## Methods

### Source and Overview of Strains

We selected 107 RR-TB strains harboring 34 unique *rpoB* mutations, including 30 within the RRDR (Multimedia Appendix 1), from the Belgian Coordinated Collections of Microorganisms and the World Health Organization Tropical Disease Research mycobacteria collection in the Institute of Tropical Medicine, Antwerp, Belgium. We prepared thermolysates of these strains as previously described, stored them at –20°C [14], and subjected them to RDTs, namely Xpert MTB/RIF, GenoType MTBDR*plus* v2.0, and Genoscholar NTM + MDRTB II.

### Xpert MTB/RIF Assay G4 Version 5

We measured the DNA concentration of the thermolysates through an Invitrogen Qubit 2.0 Fluorometer (Thermo Fisher Scientific, Waltham, MA, USA) following the manufacturer’s instructions. The calculated weight of DNA per *M tuberculosis* bacillus served as the divisor for each DNA concentration, the quotient of which was the equivalent colony-forming units (CFUs) per milliliter of each thermolysate.

We subjected thermolysates with initial concentrations of 10^8^ to 10^9^CFU/mL to 1:2 and 10-fold serial dilutions until we obtained 10^6^ to 10^7^CFU/mL, respectively. We then dispensed a 1:2 mixture of diluted thermolysate and sample reagent in the Xpert MTB/RIF cartridge following the manufacturer’s instructions.

### GenoType MTBDR*plus* v2.0 and Genoscholar NTM + MDRTB II

We subjected undiluted thermolysates to the amplification and hybridization steps of GenoType MTBDR*plus* v2.0 and Genoscholar NTM + MDRTB II following the manufacturers’ instructions.

### Analysis and Visualization of Rapid Diagnostic Test Data

We validated RDT results against the strains’ *rpoB* gene sequences, allowing for correlation between probe reactions and specific *rpoB* mutations. We then performed a comprehensive comparison and analysis of *rpoB* mutants and associated RDT results using the consensus numbering system based on *M tuberculosis* strain H37Rv [2]. We visualized results of the analysis using Geneious version 10.1.2 (Biomatters Limited) [15] and Affinity Designer 1.5.3 (Serif (Europe) Ltd) [16]. The percentage average reproducibility of the RDTs was analyzed through mutation profiles found in more than one strain.

## Results

All strains with mutations in the RRDR were correctly identified as rifampicin resistant, except for one strain with the S428R+H445R mutation that was not initially detected as *M tuberculosis* by Xpert MTB/RIF but was confirmed as rifampicin-resistant *M tuberculosis* on retesting; a strain with the D435F mutation and another strain with the L430P mutation were identified as rifampicin susceptible by GenoType MTBDR*plus* v2.0 even after a repeat test. On the probe level, each test appeared to be reproducible, ranging between 87.9% and 97.8% (Multimedia Appendix 2). Among the RDTs, Genoscholar NTM + MDRTB II yielded the highest concordance between coverage claimed by manufacturers and that observed experimentally.

The alignment of mutant and wild-type RRDR sequences in Multimedia Appendix 1 shows the nucleotide and amino acid change for each mutation profile captured by *rpoB* sequencing.

**Figure 1:**
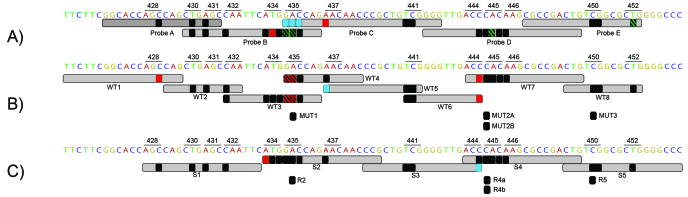
Overview of rifampicin-resistant tuberculosis rapid diagnostic test (RDT) probe reactions. The observed results for each rifampicin resistance–determining region mutation are overlaid on claimed probe coverage (light gray) of (A) Xpert MTB/RIF, (B) GenoType MTBDR*plus* v2.0, and (C) Genoscholar NTM + MDRTB II. Mutations yielding the expected probe reactions are in black and green, with delayed XpertMTB/RIF results in green, mutations missed by one probe but captured by another probe in blue, and mutations that were not at all captured by the RDT in red. Probe reactions overlaid on each other are in a striped pattern for greater visibility.

Multimedia Appendix 1 provides supporting information for Figure 1, a visualization of the probe reactions demonstrating the ability of the RDTs to capture the majority of documented RRDR mutations.

Figure 1 was generated based on observed probe reactions for each individual mutation. Hence, strains with double or triple mutations were scored twice or thrice. For instance, from a strain with the double mutation L430P+H445Q, the probe reaction for L430P was deduced as Xpert MTB/RIF probe A, GenoType MTBDR*plus* v2.0 probe WT2, and Genoscholar NTM + MDRTB II probe S1, whereas the probe reaction for H445Q was Xpert MTB/RIF probe D; for GenoType MTBDR*plus* v2.0, was WT7; and for Genoscholar NTM + MDRTB II, was S4. As expected, we found a strong correlation between observed and claimed probe reactions of RRDR mutations (Figure 1, black and green, represented by low-prevalence mutations L430P, D435G, S441L or S441Q, and L452P), although we noted delayed reactions in Xpert MTB/RIF, denoting partial inhibition of fluorescence of a particular molecular beacon [3], shown in green. It is interesting to note that some mutations were missed by one probe but captured by another, such as 435 mutations missed by Xpert MTB/RIF probe C but captured by probe B, and a codon 437 mutation in the line probe assays (Figure 1, blue mutations). End-probe mutations correctly identified by both probes in GenoType MTBDR*plus* v2.0 were Q432E (WT2 and WT3) and S441Q or S441L (WT5 and WT6). Critically, there were individual mutations completely missed by the RDTs, namely M434T and N437D by Xpert MTB/RIF, S428R and D435F by GenoType MTBDR*plus* v2.0, and M4343V by Genoscholar NTM + MDRTB II (Figure 1, red mutations). T444T is a silent mutation appropriately undetected by GenoType MTBDR*plus* v2.0, but it was captured by Xpert MTB/RIF and Genoscholar NTM + MDRTB II.

**Figure 2:**
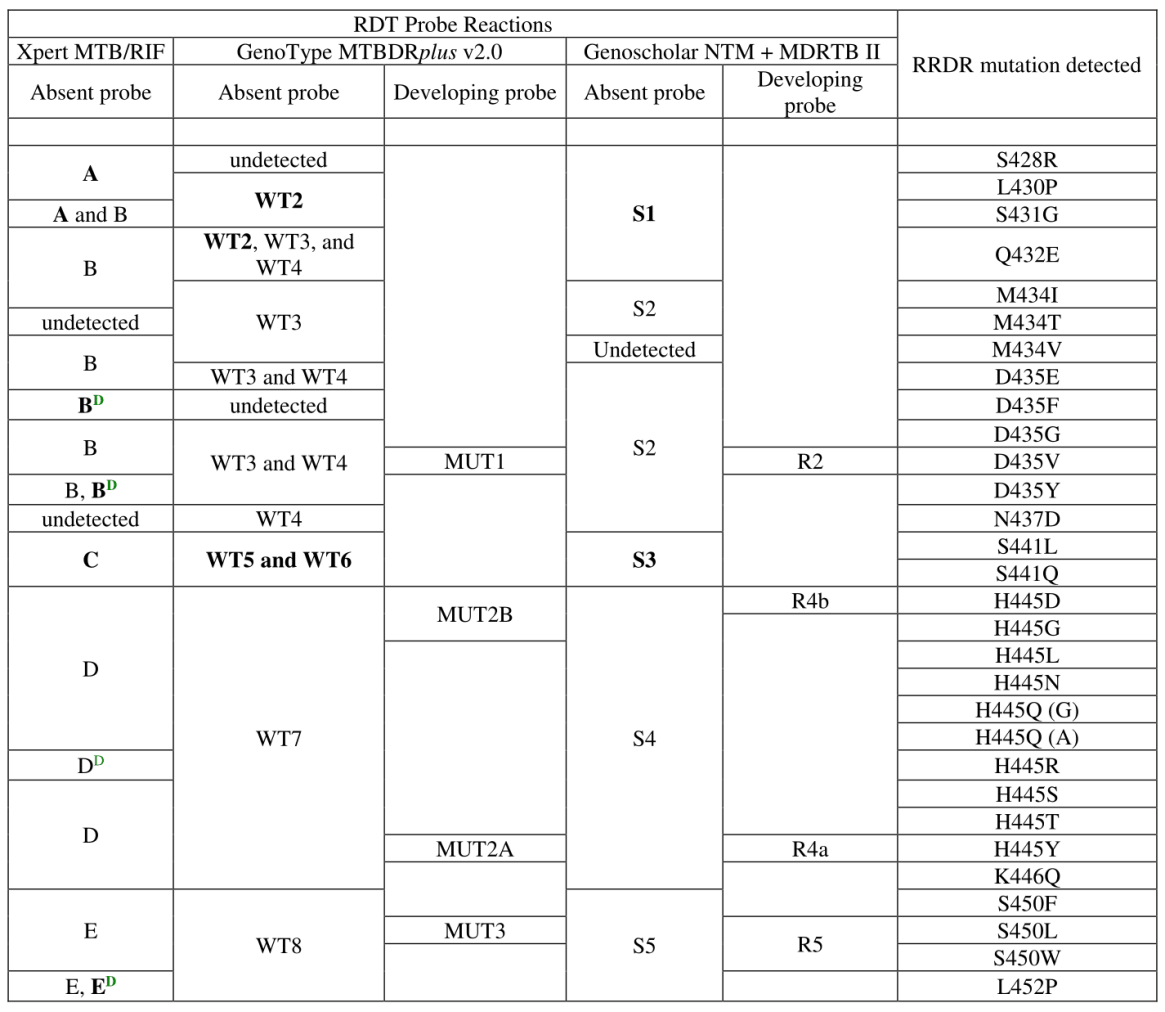
Rapid diagnostic test (RDT) probe reactions corresponding with specific rifampicin resistance–determining region (RRDR) mutations. Probe reactions with high potential use for transmission studies are bolded, while delayed probe reactions are tagged with superscript D shown in green.

We gathered the observed probe reactions representing each of the 30 RRDR mutations tested (Figure 2).

For instance, Xpert MTB/RIF probe E, GenoType MTBDR*plus* v2.0 probes WT8 and MUT3, and Genoscholar NTM + MDRTB II probes S5 and R5 correspond with mutation S450L, with the highest worldwide prevalence [17-19].

Finally, we observed a possible association of delayed Xpert MTB/RIF probe reactions with weaker fluorescence in end-probe codons and specific nucleotide substitution types. This was exemplified by guanine-to-thymine transversion in disputed mutation D435Y resulting in 53.8% of delays (average∆Ct8) for probe B and thymine-to-cytosine transition in end-probe disputed mutation L452P resulting in 25% delayed results (average∆Ct6) for probe E (Multimedia Appendices 3-8).

## Discussion

### Principal Findings and Comparison With Prior Work

Our laboratory validation of rifampicin-resistant strains and visualization of results revealed the specific relationship between unique RDT probe reactions and the majority of documented *rpoB* mutations. We also found RDT probe reactions representing low-frequency mutations in particular settings that have a high potential for use in evaluating transmission. We are proposing a novel approach to the optimal use of readily available diagnostic data through the shift to fully automated and digitized surveillance for real-time detection of RR-TB transmission hotspots.

To our knowledge, this work is the first pragmatic and scalable response to the consensus of world-renowned TB experts, published in 2016 [12], that diagnostic connectivity has the potential to eliminate TB. This is evidenced by the ability of our proposed approach to facilitate comparison of probe reactions between different RDTs used in the same setting, potentially linked to an open source software platform, by the Connected Diagnostics Initiative [12], for instance. Our findings affirm the important role of diagnostic connectivity platforms in accelerating TB control efforts and highlight the importance of fully understanding the relationship between TB diagnostic test results and drug resistance-conferring mutations. Our proposed approach will maximize the use of connected RDTs and associated data shared in repositories through a plug-in module that will automatically translate RDT results to useful information for RR-TB outbreak investigations. This will potentially aid laboratory personnel, clinicians, and national TB control programs in closing detection gaps of RR-TB cases by revealing abnormal figures or trends in particular settings, as well as real-time probable horizontal transmission and epidemiologic linkage between patients.

The comprehensive analysis of claimed and observed probe reactions revealed the ability of the RDTs to detect documented RRDR mutations. Most of the RRDR mutations analyzed yielded the expected result, concordant with manufacturers’ claims. We observed the highest concordance between observed and claimed probe coverage in Genoscholar NTM + MDRTB II, exemplified, for instance, by probe S2 detecting mutation N437D, which was missed by Xpert MTB/RIF probe C and GenoType MTBDR*plus* v2.0 probe WT5 (Figure 2,Multimedia Appendix 1, and Figure 1). Remarkably, the mutations missed by one RDT probe but captured by another were situated in the regions where the probes overlap. This affirms the role of overlapping probes in improving the usefulness of RDTs for RR-TB detection. Critically, mutations M434T missed by Xpert MTB/RIF and M434V missed by Genoscholar NTM + MDRTB II are not yet catalogued in the Tuberculosis Drug Resistance Mutation Database, nor in RefSeq [20,21]; hence, their clinical relevance is yet to be determined. Alarmingly, missed mutations, including N437D (Xpert MTB/RIF), S428R, and D435F (GenoType MTBDR*plus* v2.0), are known to confer rifampicin resistance [22]. These undetected rifampicin resistance-conferring mutations within the RRDR, together with those outside the RRDR, most notably the I491F mutation [2], may be untraceably circulated through chains of transmission, as samples with these mutations alone will be falsely classified as rifampicin susceptible, rendering treatment ineffective. Additionally, the impact of silent mutations such as T444T must be further assessed to ensure that recommended probes generate results with high specificity for rifampicin resistance. Accordingly, we suggest that manufacturers review and modify claims of mutation coverage based on the findings of this work. Additionally, for future versions of the line probe assays, manufacturers might consider synthesizing mutation probes that would also capture low-frequency mutations. These proposed modifications would improve identification of underlying mutations and thus increase the usefulness of linked RDTs for detection of rifampicin resistance and for epidemiologic surveillance.

We also gathered probe reactions representing low- to high-prevalence RRDR mutations [4,12,13,18,19,22] in the most comprehensive comparison of *rpoB* mutants and associated RDT probe reactions to date and propose their potential application for transmission studies. The use of RDT probe reactions in defined geographic settings for evaluating transmission is grounded in the need to easily deduce the underlying RRDR mutation to identify genotype clustering in time and space. When probe reactions between 2 strains correspond, even between distinct RDTs, the prevalence of probe reactions and associated RRDR mutations in a given setting must be considered when assessing the probability of a transmission event taking place. This probability will be relatively higher for probe reactions representing low-frequency mutations. This is explained by the principle that, in a normal distribution, the probability of plotting a low-frequency mutation in the tail region is low. Fitting it in the exact same location for the second time has much lower probability. Hence, when low-frequency probe reactions representing less-prevalent mutations correspond between 2 strains, an epidemiologic link might exist between the 2, and the occurrence of transmission can possibly be suspected. For example, Xpert MTB/RIF probe C, with the lowest frequency in Pakistan and Nigeria [20,21], is a potentially valid marker of S441L/Q transmission. To differentiate between the 2 mutations, RDT probe reactions must be combined with complementary genotyping test results. This proposed approach is evidenced by mutations L452P [23] and D435G [18] detected in strains epidemiologically linked to an extensively drug-resistant TB outbreak in KwaZulu-Natal, South Africa. Accordingly, we assert that Xpert MTB/RIF probes A, Bdelayed, C, and Edelayed; GenoType MTBDR*plus* v2.0 probes WT2, WT5, and WT6; and Genoscholar NTM + MDRTB II probes S1 and S3, which have a low frequency in specific settings [20,21] and detect less-prevalent mutations (emphasized in Figure 2), have a high potential for use in transmission studies. On the contrary, the probability of detecting a highly prevalent probe reaction in the distribution is high, but fitting it into the exact same point the second time would have lower probability. It would be unlikely that this is a random event solely attributable to high-frequency mutations being enriched by evolutionary convergence [24]. Therefore, when this is observed, further investigation of suspected cases of transmission is necessary. Particularly in settings of low RR-TB incidence, probe reactions corresponding with highly prevalent mutations such as Xpert MTB/RIF probes E, D, and B [20,21], if repeatedly obtained in the same health facility, may be potentially useful for finding an epidemiologic link between strains. The potential usefulness of RDT probe reactions for transmission studies lies in accounting for the frequency of probe reactions and RRDR mutations associated with RR-TB prevalence in a specific geographic setting. Linking this novel approach to a connectivity software can potentially bring about real-time reporting of suspected RR-TB transmission cases, which national TB control programs and public health officials may then investigate and confirm or exclude based on epidemiologic linking.

In addition to analyzing routine RDT data for evaluating transmission in detail, we found that RDT probe reactions corresponding with disputed mutations, supplemented with *rpoB* gene sequences linked to connectivity software, may assist in resolving discordance between phenotypic and genotypic rifampicin susceptibility results. Our observed probe reactions for disputed mutations were concordant between Xpert MTB/RIF and GenoType MTBDR*plus* v2.0, and were consistent with previous reports [13]. In contrast with discordant Xpert MTB/RIF and *Mycobacterium* growth indicator tube results due to the disputed mutation L430P [13,25], reliable probe reactions between RDTs were observed in this work. Consequently, detecting Xpert MTB/RIF probe A, GenoType MTBDR*plus* v2.0 probe WT2, or Genoscholar NTM + MDRTB II probe S1 would suggest an L430P mutation, despite obtaining a rifampicin-susceptible *Mycobacterium* growth indicator tube result. For other cases, disputed mutation D435Y can be distinguished from other variations at position 435, particularly from D435V, by a higher proportion of delayed results for probe B (Multimedia Appendix 3). Another delayed reaction observed was for probe E, associated with the disputed mutation L452P. Delayed results [3] for Xpert MTB/RIF probes B and E could be attributed to a reduced intensity of fluorescence in the end-probe codons. Alternatively, the specific nucleotide substitution, associated with the average proportion of delayed results and ∆Cts (Multimedia Appendix 3), may have caused the delays. These inferred associations may aid in better differentiation of Xpert MTB/RIF probe reactions that have a high potential use for assessing transmission and resolving discordance between phenotypic and genotypic drug susceptibility results. On the contrary, disputed mutations H445L and H445N associated with high in vitro resistance [26] could not be distinguished from undisputed mutations in codon 445; complementary *rpoB* sequences would be very beneficial in this case. We strongly suggest that each specific mutation and RDT result be assessed case-by-case for discordant results when compared with phenotypic drug susceptibility testing and their potential for transmission clustering studies.

### Scope and Limitation

The limitation of the study was the lack of strains with RRDR mutations outside labeled codon positions in Figure 1. However, to our knowledge, the uncovered mutations have not yet been encountered in clinical isolates and thus are likely to be extremely rare. This work is not a field evaluation of the potential of connected TB diagnostic data, but rather a pragmatic laboratory validation and analysis of test results.

### Conclusions

We propose a novel approach for potential real-time detection of RR-TB transmission through fully using connected TB diagnostics and a shared repository of test results. To our knowledge, this is the first pragmatic and scalable work in response to the consensus of world-renowned TB experts in 2016 on the potential of diagnostic connectivity to accelerate efforts to eliminate TB [12]. This is evidenced by the ability of our proposed approach to facilitate comparison of probe reactions between different RDTs used in the same setting.

While less-prevalent RDT probe reactions corresponding with low-frequency mutations would be most suitable for evaluating transmission, combining this approach with complementary genotyping tests such as membrane-based spoligotyping, MIRU-VNTR, deep targeted sequencing, or whole genome sequencing on cultured isolates [9] would increase the resolution of the approach in identifying RR-TB transmission hotspots. Supplementing RDT test results with spoligotyping data, for instance, not only illustrates data pooling [12], but also enhances the discriminatory power of this combined approach to confirm suspected cases of TB transmission. This proposed prospective approach, inspired by the SpoNC tool, which combined *pncA* sequencing and spoligotyping data for detection of pyrazinamide-resistant TB transmission clusters [9], will be validated against conventional approaches through strains with specific mutations and originating from documented clusters.

Integrating the combined approach as an additional module in the connectivity platform will not only automate the analysis of shared RDT results and its translation into crucial clinical and public health information, but also allow more precise real-time estimation of transmitted RR-TB proportions in a population. Detection of RR-TB transmission hotspots would initiate a timely outbreak response and appropriate investigation of suspected transmission cases based on epidemiologic linking that would prevent further spread of TB.

## Appendix

Multimedia Appendix 1 Alignment of *Mycobacterium tuberculosis* strain H37Rv and rifampicin resistance–determining region mutant sequences showing alternative nucleotides and amino acids for each mutation profile analyzed.

Multimedia Appendix 2 Reproducibility of RDT probe profiles determined through mutation profiles found in multiple strains.

Multimedia Appendix 3 Mutations (with wild-type and mutant nucleotide bases) that returned delayed result for Xpert MTB/RIF with corresponding nucleotide substitution type, capturing probe, location of codon with respect to claimed probe coverage, Ct, ∆Ct, and average ∆Ct values, and percentage of delayed result considering all strains tested.

Multimedia Appendix 4 Ct curve of mutation D435Y captured by probe B with single transversion substitution type, Ct value 27.9 and ∆Ct 6.

Multimedia Appendix 5 Ct curve of mutation D435F captured by probe B with double transversion substitution type, Ct value 38.1 and ∆Ct 16.8.

Multimedia Appendix 6 Ct curve of mutation D435V captured by probe B with single transversion substitution type, Ct value 38.6 and ∆Ct 17.

Multimedia Appendix 7 Ct curve of mutation H445R captured by probe D with single purine transition substitution type, Ct value 27.5 and ∆Ct 8.2.

Multimedia Appendix 8 Ct curve of mutation L452P captured by probe E with single pyrimidine transition substitution type, Ct value 24.5 and ∆Ct 6.

## Abbreviations

CFU: colony-forming unit
MDR-TB: multidrug-resistant tuberculosis
RDT: rapid diagnostic test
RRDR: rifampicin resistance–determining region
RR-TB: rifampicin-resistant tuberculosis
TB: tuberculosis
