# Supplementary figures and images for: Potential Application of Digitally Linked Tuberculosis Diagnostics for Real-Time Surveillance of Drug-Resistant Tuberculosis Transmission: Validation and Analysis of Test Results
Source: JMIR Med Inform. 2018 Feb 27;6(1):e12. doi: 10.2196/medinform.9309 (PMC5849801; doi:10.2196/medinform.9309)

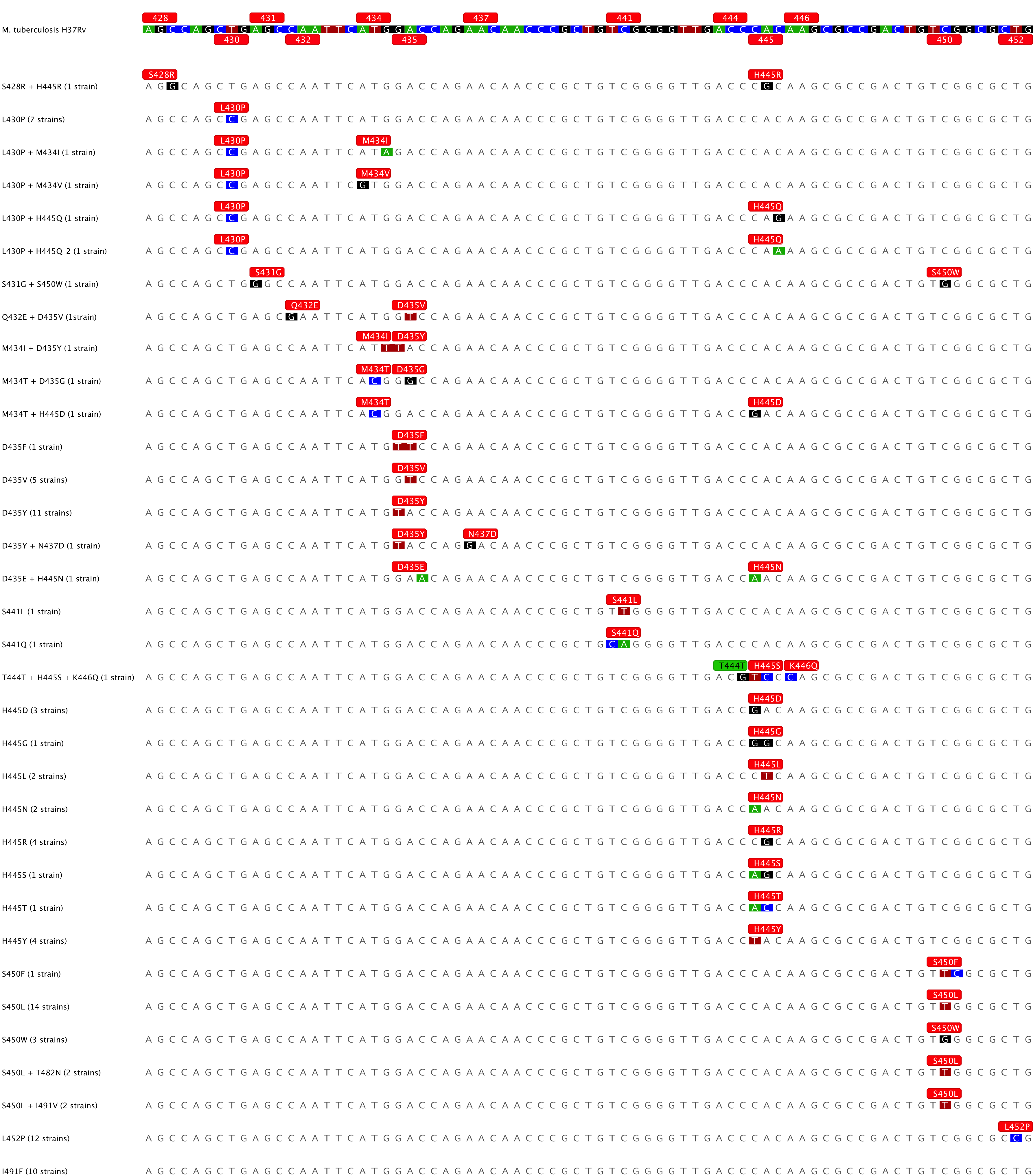

Supplement: Multimedia Appendix 1 [file medinform_v6i1e12_app1.png]
