# Supplementary material for: Potential Application of Digitally Linked Tuberculosis Diagnostics for Real-Time Surveillance of Drug-Resistant Tuberculosis Transmission: Validation and Analysis of Test Results
Source: JMIR Med Inform. 2018 Feb 27;6(1):e12. doi: 10.2196/medinform.9309 (PMC5849801; doi:10.2196/medinform.9309)
Supplement: Multimedia Appendix 2 [file medinform_v6i1e12_app2.pdf]

Multimedia Appendices

Multimedia Appendix 1. Reproducibility of RDT probe profiles determined through mutation profiles found in multiple strains.

**RDT**	**Number of mutation profiles**	**Average percent reproducibility**
XpertMTB/RIF	11	87.94
MTBDR*plus*v2.0	11	96.31
GenoscholarNTM+MDRTBII	11	97.78
