# Supplementary material for: Potential Application of Digitally Linked Tuberculosis Diagnostics for Real-Time Surveillance of Drug-Resistant Tuberculosis Transmission: Validation and Analysis of Test Results
Source: JMIR Med Inform. 2018 Feb 27;6(1):e12. doi: 10.2196/medinform.9309 (PMC5849801; doi:10.2196/medinform.9309)
Supplement: Multimedia Appendix 3 [file medinform_v6i1e12_app3.pdf]

Multimedia Appendices

Multimedia Appendix 2. Mutations (with wildtype and mutant nucleotide bases) which returned delayed result for XpertMTB/RIF with corresponding nucleotide substitution type, capturing probe, location of codon with respect to claimed probe coverage, Ct, Δ Ct, and average Δ Ct values, and percentage of delayed result considering all strains tested.

Mutation	Nucleotide Substitution Type	Probe	Location of codon	Ct	Δ Ct	Average Δ Ct	Proportion
(GAC) D435Y (TAC)	Transversion guanine to thymine	B	towards end of Probe B	29.5	4.7	8	53.8
				24.5	5.9		
				27.9	6		
				23.9	7.2		
				24.5	8.2		
				25.6	9		
				25.2	9.6		
(GAC) D435F (TTC)	Double transversion guanine adenine to thymine thymine			38.1	16.8	16.8	sole strain
(GAC) D435V (GTC)	Transversion adenine to thymine			38.6	17	17	14.3
(CAC) H445R (CGC)	Transition adenine to guanine	D	around middle region of Probe D	27.5	8.2	9.87	80
				32.7	9.9		
				25.9	11.5		

(CTG) L452P (CCG)	Transition<br><br>thymine to cytosine	E	end of Probe E	26.6	5.2	6	25
				24.5	6		
				26.5	7.1		

6
