# Supplementary material for: Potential Application of Digitally Linked Tuberculosis Diagnostics for Real-Time Surveillance of Drug-Resistant Tuberculosis Transmission: Validation and Analysis of Test Results
Source: JMIR Med Inform. 2018 Feb 27;6(1):e12. doi: 10.2196/medinform.9309 (PMC5849801; doi:10.2196/medinform.9309)
Supplement: Multimedia Appendix 5 [file medinform_v6i1e12_app5.pdf]

Multimedia Appendices

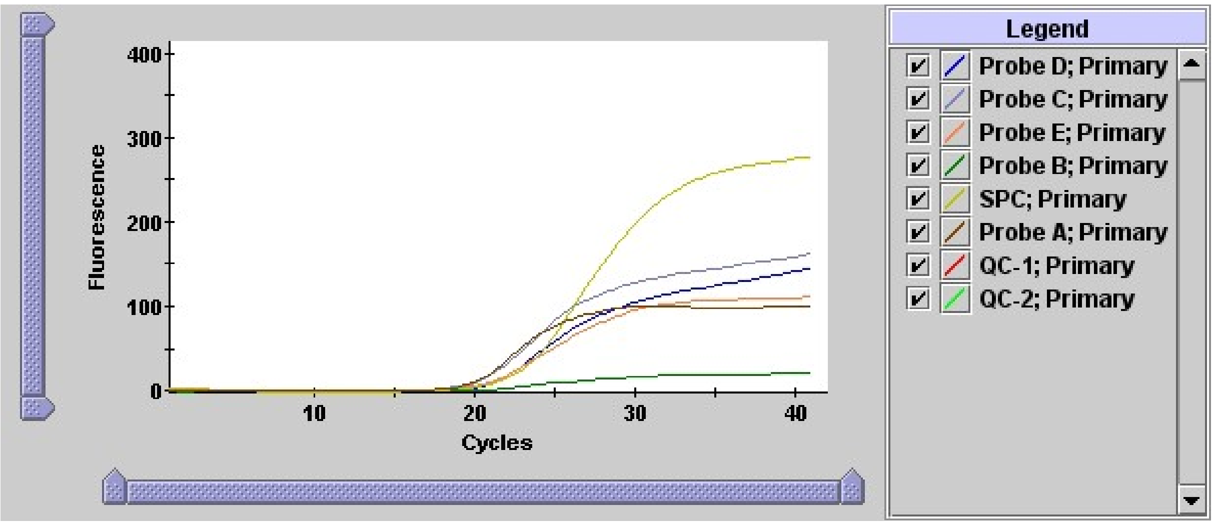

Multimedia Appendix 4. Ct curve of mutation D435F captured by probe B with double transversion substitution type, Ct 38.1 and ΔCt 16.8.
